# Effectiveness and cost-effectiveness of a community-based mental health care programme (GBV) for people with severe mental illness in Germany: study protocol for a randomised controlled trial

**DOI:** 10.1186/s13063-020-04492-y

**Published:** 2020-06-30

**Authors:** Annabel Sandra Mueller-Stierlin, Friedrich Meixner, Anne Kohlmann, Mara Schumacher, Anke Hänsel, Melanie Pouwels, Nicole Bias, Sabrina Hartl, Jessica Reichstein, Elke Prestin, Nils Greve, Thomas Becker, Reinhold Kilian

**Affiliations:** 1 Department of Psychiatry II, Ulm University, Bezirkskrankenhaus Günzburg, Günzburg, Germany; 2 Institute of Epidemiology and Medical Biometry, Ulm University, Ulm, Germany; 3 Dachverband Gemeindepsychiatrie, Cologne, Germany

**Keywords:** Community-based mental health care, Cross-sectoral care, Assessment, Severe mental illness, Depression, Schizophrenia, Empowerment, Cost-effectiveness

## Abstract

**Background:**

The community-based mental health care programme GBV is based on the British Community Mental Health Teams and the Dutch Flexible Assertive Community Treatment model. In addition, the programme offers crisis-intervention services. A special feature of this integrated care programme is the initial standardised assessment process regarding empowerment, unmet care needs, and psychosocial functioning, used to verify the need for such a comprehensive form of care. The project evaluates the assessment process and analyses the effectiveness and cost-effectiveness of GBV compared to treatment as usual.

**Methods:**

This randomised, controlled study includes five assessments over 2 years. In twelve regions in Germany, 1000 patients with severely impaired psychosocial functioning and unmet care needs will be recruited. Study eligibility relies on an indication for GBV based on the results of the initial assessment. The primary outcome is improved self-reported empowerment. Further outcomes include improved treatment satisfaction and subjective quality of life, reductions in patients’ unmet needs and illness-related clinical and social impairment, and an improved cost-effectiveness ratio of the resources used (from the perspectives of both statutory health insurance and the national economy). In addition, the GBV’s effects on the burden and quality of life of informal caregivers of patients will be investigated.

**Discussion:**

The study’s results are expected to provide information on whether the community-based mental health care programme GBV contributes to improving mental health care provision in Germany. In addition, the study will show whether the GBV successfully overcomes the weaknesses that former research has identified regarding a German integrated care programme. Such improvement is particularly expected with respect to the semi-structured assessment within GBV.

**Trial registration:**

German Clinical Trial Register, DRKS00019086. Registered on 3 January 2020.

## Administrative information

Note: the numbers in curly brackets in this protocol refer to SPIRIT checklist item numbers. The order of the items has been modified to group similar items (see http://www.equator-network.org/reporting-guidelines/spirit-2013-statement-defining-standard-protocol-items-for-clinical-trials/).

**Table:** 

Title {1}	Effectiveness and cost-effectiveness of a community-based mental health care programme (GBV) for people with severe mental illness in Germany: study protocol for a randomised controlled trial
Trial registration {2a and 2b}.	German Clinical Trial Register DRKS00019086 Item 2b is met
Protocol version {3}	03/04/2020 Version 1.0
Funding {4}	This study is funded by the Innovation Fund of the Federal Joint Committee (G-BA), grant number 01NVF18028 (GBV).
Author details {5a}	Dr Annabel Sandra Mueller-Stierlin,^1,2^ Friedrich Meixner,^1^ Anne Kohlmann,^1^ Mara Schumacher,^1^ Anke Hänsel,^1^ Melanie Pouwels,^1^ Nicole Bias,^1^ Sabrina Hartl,^1^ Jessica Reichstein,^3^ Dr Elke Prestin,^3^ Nils Greve,^3^ Prof Dr Thomas Becker,^1^ Prof Dr Reinhold Kilian^1^ ^1^ Department of Psychiatry II, Ulm University, Bezirkskrankenhaus Günzburg, Günzburg, Germany ^2^ Institute of Epidemiology and Medical Biometry, Ulm University, Ulm, Germany ^3^ Dachverband Gemeindepsychiatrie, Cologne, Germany.
Name and contact information for the trial sponsor {5b}	Nils Greve, Dachverband Gemeindepsychiatrie, Cologne, Germany. Phone: +49 163 2482112, mail: greve@psychiatrie.de
Role of sponsor {5c}	The sponsor initiated the trial and was involved in designing the study. Representatives of the sponsor—NG, EP, and JR—belong to the trial steering committee. The sponsor will not be involved in the collection, management, analysis, or interpretation of the data, or in the decision to submit the report for publication.

## Introduction

### Background and rationale {6a}

Beyond controlling psychiatric symptoms and ensuring the satisfaction of elementary needs, contemporary psychiatric services are expected to support individual recovery and comprehensive social inclusion among their main targets [1]. For this purpose, the World Health Organization considers the provision of comprehensive, integrated, and responsive mental health and social care services in community-based settings as the universal state of the art of mental health care systems [2].

As a country, Germany has one of the highest expenditures on mental health care in the world, in terms of population and gross domestic product. The German mental health care system is characterised by a well-developed system of psychiatric hospitals and psychiatric departments at general hospitals providing in- and outpatient treatment of patients with severe mental illness [3]. Psychiatric outpatient treatment of people with common mental disorders is mainly provided by psychiatrists and psychological psychotherapists working in private practice. Access to mental health care, including medication, is guaranteed under social law by the statutory health insurance scheme, which covers about 90% of the German population [3]. In comparison to this medical service-based mental health care system, community mental health care (with a psychosocial focus) is underdeveloped in most parts of Germany. Beyond historical developments in the German health and social care system, the current disparities in the provision of community mental health care mainly exist because, in contrast to medical care, the responsibility for providing and financing community care is at the level of the federal states and the communities [4, 5]. This fragmentation of the provision and financing of mental health care is suspected to have caused several shortcomings regarding the effectiveness and efficiency of mental health care in Germany. In particular, many experts criticise the lack of systematic coordination between in- and outpatient care resulting in the discontinuation of treatment, which in turn leads to unnecessary inpatient admissions and increases the risk of social and occupational disintegration due to mental disorders [5].

Several changes to the social code have recently been implemented to overcome the fragmentation of mental health care provision, thus extending the possibilities for the common provision and financing of medical and community mental health services. With regard to developments in other countries, over the last decade, various pilot projects have been conducted in Germany to implement and evaluate different approaches for integrating medical- and community-based mental health care. The results of these studies are inconclusive with regard to the assessed effectiveness and efficiency of integrated mental health care approaches but also with regard to the generalisability of their outcomes [6–11].

This is mainly because, with one exception [8], none of these projects implemented evidence-based approaches to integrated mental health care, such as assertive community treatment or intensive case management. A majority of the projects used certain components, like case management or multiprofessional treatment, which were provided in addition to standard care. In most cases, these additional treatment components were financed on the basis of lump sums, in contrast to the usual fee-for-service payment in the German health care system.

Moreover, to date, no German study has examined the effectiveness and efficiency of integrated mental health care compared to care as usual (CAU) in a randomised controlled trial (RCT) design. Consequently, mental health care decision-makers in Germany still lack an empirical basis by which to assess the advantages and disadvantages of implementing integrated mental health services into routine psychiatric treatment.

In a recent study, we revealed that the additional provision of integrated mental health care components based on case management to people with mental illness, on the whole, was not more effective than CAU with regard to the primary outcome (empowerment) and most of the secondary outcomes (psychosocial impairment, met needs, and satisfaction). We suspected that this result was mainly because the services provided were insufficiently tailored to the individual needs of the service users [10].

In the current study, a new approach to community-based integrated mental health care, called Gemeindepsychiatrische Basisversorgung (GBV), will be evaluated using a RCT design. GBV includes an initial need assessment and an individually tailored mental health care plan as core elements of an integrated mental health care process. All relevant medical and social services are integrated and coordinated in close consultation with the service users and their family, or other persons to whom they feel close [12, 13].

### Objectives {7}

The aims of this study are to examine the adequate implementation, effectiveness, and cost-effectiveness of the community-based mental health care programme GBV for people with severe mental illness in Germany.

The following hypotheses will be examined individually:

- Community-based mental health institutions can integrate GBV into regular mental health care for people with severe mental illness.
- The indications for GBV can be assessed validly and reliably based on objective criteria.
- Compared to CAU, the use of GBV leads to a stronger empowerment effect, made apparent by an improved subjective ability to live a self-determined and self-reliant way of life, independently arrange social relationships, actively participate in mental health care, and have increased expectations of self-efficacy and future expectations (primary outcome criterion).
- Compared to CAU, the use of GBV leads to a stronger improvement of subjective quality of life as well as reductions to unmet care needs and the clinical and social impairment of patients related to their illness.
- Compared to CAU, the use of GBV leads to a greater reduction in the burden on informal caregivers and to improvements in their quality of life.
- From a health economic perspective, the use of GBV improves the cost-effectiveness ratio of the resources used, compared to CAU.
- From the perspective of the statutory health insurance, the implementation of GBV does not lead to a significant increase in expenditures.

### Trial design {8}

The present study is a multisite RCT involving people with severe mental illness and, if available, their informal caregivers, with five measurement points at 6-month intervals. The participants are randomly assigned to either the GBV or CAU group, with a 1:1 allocation. The study is primarily aimed at determining the superiority of GBV over CAU. Exploratory analysis includes superiority analysis of GBV over CAU for secondary outcomes, equivalence testing of GBV and CAU for costs of statutory health insurance agencies, and descriptive analyses.

## Methods: participants, interventions, and outcomes

### Study setting {9}

The study will take place in five federal states of Germany, in cooperation with twelve local community mental health care providers. The list of local service providers is available online (https://gbv.online/kontakt/regionale-ansprechpartnerinnen/). The intervention is based on contracts for model projects according to § 64b SGB V, which were concluded between the health insurance companies involved in the project and the local service providers.

### Eligibility criteria {10}

The inclusion criteria for participants are as follows:

- Minimum age: 18 years
- Presence of mental illness in ICD-10 diagnosis groups F2, F3, F4, F5, or F6
- Membership in a participating health insurance company
- Permanent residence in one of the twelve defined project regions
- Indications for GBV established by a screening assessment

Within a structured assessment, the local GBV teams will assess the indications for GBV in consultation with a physician or psychotherapist. The following standardised scales will be applied as part of the assessment:

- The Assessment of Empowerment in Patients with Affective and Schizophrenic Disorders (EPAS), to determine the participants’ self-assessed ability to lead a self-determined and responsible way of life.
- The Camberwell Assessment of Need (CAN), to determine unmet needs, which is an external assessment by members of the GBV team.
- The Health of the Nation Outcome Scale (HoNOS), to determine patients’ clinical and social impairment due to illness, which is an external assessment by members of the GBV team.

The cut-off values for the three scales were derived from data from a previous study on integrated mental health care in Germany [10]. These cut-offs are ≤ 3.3 for mean EPAS total score, ≥ 4 for CAN unmet needs (sum score), and ≥ 12 for HoNOS total sum score.

Based on the results of these scales and upon consideration of the clinical evaluation in consultation with a physician or psychotherapist, the following decision regulations will be applied:

- Within cut-off values for all scales: no indication for GBV
- Outside cut-off values for one or two scales: indication only with a clinical recommendation
- Outside cut-off values for all three scales: indication for GBV

The exclusion criteria for participants are as follows:

- Primary clinical diagnosis in ICD-10 groups F0, F1, F7, F8, or F9
- Participation in other integrated mental health care programmes in the last 6 months before recruitment

### Who will take informed consent? {26a}

At the beginning of the assessment, local community mental health care providers will provide the patients with verbal and written information regarding the study and ask the patients to give written informed consent if they agree to participate in this study.

The patients will be asked to nominate an informal caregiver who can be interviewed with patient approval. The nominated informal caregivers will receive the written study information and will be invited to contact the local research associate if they have any remaining questions. The informal caregivers’ participation requires their written consent.

### Additional consent provisions for collection and use of participant data and biological specimens {26b}

Informed consent includes consent for using data from the statutory health insurance.

## Interventions

### Explanation for the choice of comparators {6b}

The range of offerings for participants in the control group (CAU) is restricted to standard care. Participants in the control group have no access to the GBV’s specific services. The current standard mental health care in Germany is mainly provided by psychiatric clinics and day clinics as well as psychiatrists and psychotherapists in private practice. Vocational rehabilitation centres, community mental health care centres, and various residential and nursing facilities offer a wide range of nonmedical professional and psychosocial services. Crisis support teams have so far been implemented in only few regions of Germany, although they are now required by law in Bavaria, based on the Bavarian Mental Health Assistance Act (BayPsychKHG). In sum, mental health service offerings are wide-ranging but vary from place to place.

### Intervention description {11a}

Participants in the intervention group will receive GBV in addition to routine care. GBV comprises the coordination and provision of community-based mental health care through multiprofessional GBV teams involving a physician. GBV is modelled on the British Community Mental Health Teams and the Dutch Flexible Assertive Community Treatment model in consideration of the S3 guideline Psychosocial Therapies for Severe Mental Illnesses by the German Society of Psychiatry and Psychotherapy, Psychosomatic Medicine and Neurology (DGPPN) [1].

#### Case management

A team member will act as a case manager for the participant and maintain continuous contact with him/her at a frequency appropriate to his/her needs. In addition, the case manager will organise network meetings based on systemic concepts between the patient, his/her informal caregivers, and all service providers involved to coordinate and harmonise their services.

#### Service planning

An individual mental health service plan will be developed with each participant on the basis of the participant’s needs identified at the initial assessment. Network meetings involving patients, service providers, and informal caregivers or other related persons will be used to review and adjust the mental health service planning as required but at least every 6 months.

#### Crisis services

A locally organised four-level crisis service will be available around the clock to the participants of the intervention group. It comprises a telephone hotline staffed by psychosocial specialists, an equally staffed crisis-intervention team, a crisis home that can be occupied and is staffed at all times, and a medical service that can be contacted by telephone around the clock and can be attended, if necessary.

### Criteria for discontinuing or modifying allocated interventions {11b}

GBV is a complex mental health care programme and follows a need-adapted approach. Consequently, the intervention varies between patients and over time depending on the patients’ current mental health state and preferences.

### Strategies to improve adherence to interventions {11c}

All GBV teams participate in multicentre training activities in order to ensure the standardisation of key features of the intervention.

The GBV concept includes psychosocial measures, such as relationship building with the case manager and considering patient preferences, which are expected to improve adherence to the intervention.

### Relevant concomitant care permitted or prohibited during the trial {11d}

There are no restrictions regarding concomitant care during the trial.

### Provisions for post-trial care {30}

Post-trial care is not planned, as it is not expected that anybody will suffer harm from trial participation. However, the concluding phase (months 19 to 24) of the GBV interventions is aimed at transferring the client to CAU.

### Outcomes {12}

All outcomes will be assessed over 24 months at 6-month intervals: *t*_0_, *t*_0_ + 6 months (*t*_1_), *t*_0_ + 12 months (*t*_2_), *t*_0_ + 18 months (*t*_3_), *t*_0_ + 24 months (*t*_4_).

The primary outcome is change in empowerment (EPAS, mean total score) [14] over 24 months. This outcome was chosen because contemporary care for people with permanent severe mental illness is aimed not only at controlling disease symptoms but also at empowering patients by increasing their capacities for a largely independent lifestyle and comprehensive social and professional inclusion.

The secondary outcomes include changes over 24 months in (1) satisfaction with psychiatric treatment using the Questionnaire of Treatment Satisfaction (ZuF8, sum score) [15, 16], (2) subjective quality of life using the World Health Organisation Quality of Life—short version (WHO-QoL-BREF, mean score rescaled to the range between 0 and 100) [17], (3) count of unmet needs for psychiatric and psychosocial services using the Camberwell Assessment of Need—European version (CAN) [18, 19], and (4) psychosocial and clinical impairment using the Health of the Nation Outcome Scale (HoNOS, sum score) [20–23]. Furthermore, the cost-utility of the GBV treatment in comparison to CAU will be investigated from the societal perspective and from the perspective of the statutory health insurance (payer perspective). Costs will be estimated from the view of the statutory health insurance and of the national economy using the Client Sociodemographic and Service Receipt Inventory (CSSRI) [24]. The cost-utility ratio is defined as the costs of gaining a “healthy” year of life (QALY), measured by the Euro Quality of Life—5 dimensions questionnaire (EQ-5D) [25].

The secondary outcomes assessed in informal caregivers will be changes over 24 months in (1) the perceived burden related to support of patient suffering from serious mental illness using the Involvement Evaluation Questionnaires (IEQ-EU, sum score) [26], (2) satisfaction with the patient’s psychiatric treatment using the ZuF8 (sum score) [15, 16], and (3) subjective quality of life using the WHO-QoL-BREF (mean score rescaled to the range between 0 and 100) [17].

The participants are asked to immediately report serious adverse events. Inpatient stays will be recorded at the next follow-up assessment, at the latest, by completing the CSSRI.

### Participant timeline {13}

The start of patient recruitment is set for June 1, 2020, and is to be completed within 12 months. Recruitment of and data collection from informal caregivers will take place simultaneously. The individual participation period in the study will be 24 months. In both study groups, baseline and four follow-up assessments will take place at 6-month intervals (*t*_0_, *t*_0_ + 6 months [*t*_1_], *t*_0_ + 12 months [*t*_2_], *t*_0_ + 18 months [*t*_3_], and *t*_0_ + 24 months [*t*_4_]). The follow-up windows are defined as ± 4 weeks for the intended time point. The last data collection of the last study participant should be completed by June 1, 2023 (last patient out [LPO] planned).

The following study schemes depict the course of the study (Tables 1 and 2). GBV team members of the local community mental health providers will enrol patients (including providing informed consent and screening), while evaluation team members at Ulm University will enrol informal caregivers and collect the data.

**Table 1:**
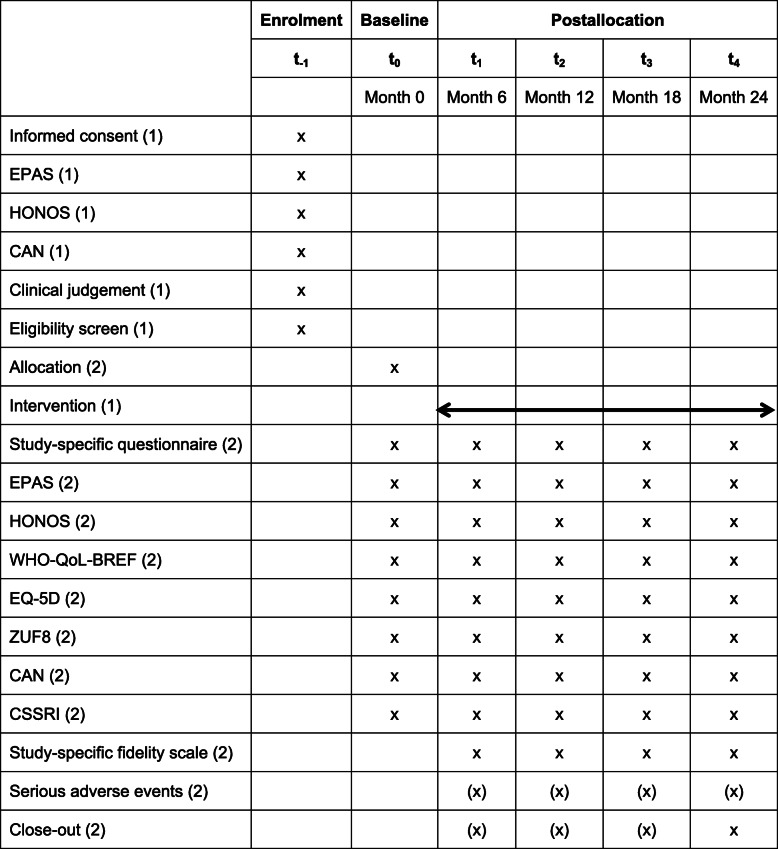
Patient enrolment, intervention, and assessment schedule

The activities will be conducted either by (1) GBV team members or (2) evaluation team members

**Table 2:**
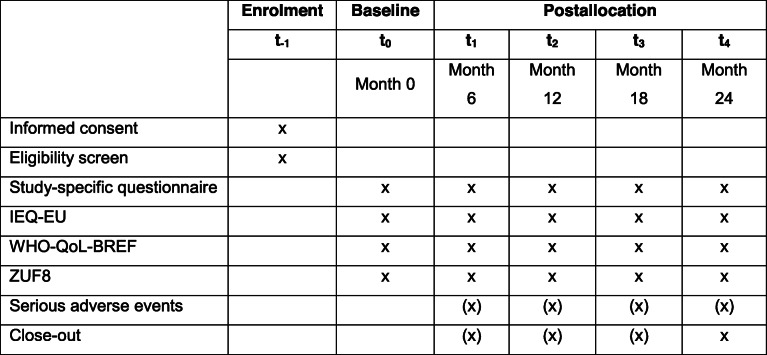
Informal caregiver enrolment and assessment schedule

All activities will be conducted by evaluation team members

The intervention will start immediately after *t*_0_ is completed and participants are assigned to the intervention group. Therefore, a GBV team member will join the research worker and the participant at the end of the baseline assessment. If the participant is assigned to the intervention group, the GBV team member will schedule an initiation meeting with the participant. If the participant is assigned to the CAU group, the GBV team member will inform the client about other regional services, as would be expected for CAU.

### Sample size {14}

The calculation of the expected effect size is based on the results of a previous study (IVPOWER) [10]. Since the total score of the scale for measuring empowerment in patients with affective and schizophrenic disorders (EPAS) as the primary outcome proved to be rather difficult and slow to change, a low effect size of 0.20 and a high power of 0.90 were chosen for the sample size calculation of the present study.

The sample size calculation was based on a linear mixed-effects regression model with an interaction effect between study group and measurement time. The expected effect size, alpha value, beta value, number of measurements, number of study sites, and expected number of dropouts over all follow-up measurements were taken into account.

Based on the results of the IVPOWER study, an increase in the effect size of the primary outcome parameter is expected, from 0 standard deviations (SD) at baseline (*t*_0_) to 0.2 SD at the last assessment (*t*_4_). Furthermore, a decreasing dropout rate of 15% between *t*_0_ and *t*_1_, 7% between *t*_1_ and *t*_2_, 4% between *t*_2_ and *t*_3_, and 2% between t_3_ and t_4_ is expected, so that the expected sample size at *t*_4_ still comprises 72% of the initial sample.

A sample size calculation using the Web-based programme RMASS (www.rmass.org) yielded a number of at least 978 subjects for twelve study sites. On this basis, the target sample size was set at 1000 participants.

We expect to include about 350 informal caregivers, of whom about 225 will complete the study based on the experience gained in the IVPOWER study (recruitment rate = 36.8%; dropout rate = 35.6%).

### Recruitment {15}

Various service providers in the regional mental health care system and the participating health insurance agencies will inform the study participants about the project. The information will be provided in personal interviews, via telephone, and via written information.

All patients included in the study will be asked to name an informal caregiver with whom he/she is in regular contact and who supports him/her in coping with his/her illness.

## Assignment of interventions: allocation

### Sequence generation {16a}

In order to avoid selection bias, random allocation into the two study groups—GBV and CAU—will be undertaken. Random assignment to the control or intervention group with a 1:1 allocation will occur using the ROM software [27] according to a computer-generated randomisation schedule stratified by site using permuted blocks of random sizes. The block sizes will not be disclosed, to ensure concealment.

### Concealment mechanism {16b}

The Institute of Epidemiology and Medical Biometry at Ulm University will carry out the randomisation, guaranteeing the independence of the group allocation from the data collection and analysis processes.

All participants who give consent to participation and fulfil the inclusion criteria will be randomised. For this, GBV teams will contact the local research worker who will schedule the baseline assessment (*t*_0_) and request randomisation by e-mail from the Institute of Epidemiology and  Medical Biometry, Ulm University, Germany. The randomisation form includes the participant ID (including his/her study ID), date of informed consent, and approval of eligibility. The requesting research worker will obtain a response by mail within one working day. Allocation disclosure will occur after the baseline assessment is completed, with the attendance of the participant, the research worker, and a member of the GBV team.

### Implementation {16c}

The Institute of Epidemiology and Medical Biometry at Ulm University will generate the allocation sequence. The GBV teams will enrol participants and inform the research workers at Ulm University about the participants’ enrolment. The research workers will initiate randomisation, collect baseline data, and assign the participants to interventions.

## Assignment of interventions: blinding

### Who will be blinded {17a}

Due to the type of intervention, blinding of the participants and service providers is not feasible. Furthermore, research workers responsible for data collection cannot be blinded, since they would have been unblinded at the latest when documenting GBV-specific services in the CSSRI and when collecting the data for process evaluation. The same applies for all researchers involved in data monitoring and data management. Thus, only the researcher performing the main analysis will be blinded until analysis has been completed.

### Procedure for unblinding if needed {17b}

Group affiliation of study participants will be revealed to the statistician by the data manager on duty after the finalisation of the main data analysis.

## Data collection and management

### Plans for assessment and collection of outcomes {18a}

Screening will take place at local mental health care facilities, and GBV team members will collect the data in paper-based form. The local research worker will collect further data (from *t*_0_ to *t*_4_) at a location of the study participant’s choosing, primarily in electronic form. If electronic data collection is not possible, the data will be collected on paper and electronically transferred later. Informal caregivers will be asked to fill out paper-based questionnaires on their own and send them by mail to the coordinating centre. Centralised electronic data capture is intended for these questionnaires. If necessary, specific issues will be clarified by phone.

Study-specific questionnaires will be compiled for sociodemographic data, medical history, living conditions, and social life. At the follow-up assessments (*t*_1_, *t*_2_, *t*_3_, and *t*_4_), a study-specific fidelity scale will be used to assess interventional compliance among the service providers and to gain ancillary information about the importance of and the satisfaction with local community mental health services from the patients’ perspective. In addition, all local GBV providers will be asked to answer a catalogue of quality criteria for community mental health care as defined on the basis of the  S3 guideline “Psychosocial Therapies for Severe Mental Illnesses” of the DGPPN [1] in order to check for adequate implementation of GBV. Standardised questionnaires will be used to assess further process variables such as interprofessional collaboration and recovery orientation.

Only standardised questionnaires with known and at least satisfactory psychometric characteristics will be used to assess the outcomes:

- Assessment of Empowerment in Patients with Affective and Schizophrenic Disorders (EPAS) [14]
- Questionnaire of Treatment Satisfaction (ZUF8) [15, 16]
- World Health Organisation Quality of Life—short version (WHO-QoL-BREF) [17]
- Camberwell Assessment of Need—European version (CAN) [18, 19]
- Health of the Nation Outcome Scale (HoNOS) [20–23]
- Euro Quality of Life—5 dimensions (EQ-5D) [25]
- Client Sociodemographic and Service Receipt Inventory (CSSRI) [24]
- Involvement Evaluation Questionnaire (IEQ-EU) [26]

The primary outcome will be measured by means of the EPAS, which measures empowerment as the patient’s perceived possibilities to control his or her own life on five dimensions: daily living, social relationships and sexuality, psychiatric treatment, hope and self-efficacy and self-esteem. This self-assessment instrument has 33 items and five additional items each for patients who are employed and for patients with minor children. Cronbach’s *α* = 0.94 was obtained for the total scale [14]. Originally developed to assess empowerment in patients with affective and schizophrenic disorders, the EPAS has also proved to be reliable in patients with other types of severe mental disorders [10, 28].

The German short version of the Client Satisfaction Questionnaire (CSQ8), published as Fragebogen zur Patientenzufriedenheit (ZUF8) [15, 16], is a suitable tool for measuring patient satisfaction with their mental health care.

The WHO-QoL-BREF captures the patients’ subjective quality of life in the dimensions of physical health, mental well-being, social relationships, and environmental conditions. The WHO-QoL-BREF is a self-assessment tool with 25 items [17].

The CAN records patients’ perceived needs and the extent to which they are met, covering 23 areas. No special knowledge is required to work with this instrument [18, 19].

The HoNOS measures the clinical and psychosocial impairment of patients in twelve dimensions, independent of diagnosis. After an appropriate briefing, members of all mental health professions can carry out the HoNOS assessment [20–23].

The EQ-5D enables the assessment of health conditions as a basis for determining quality-adjusted life years (QALY) [25].

The use of health and psychosocial care services will be recorded using the CSSRI [24]. Health care costs are estimated by multiplying the service units used by the billing costs per unit for a 6-month period. For the health economic analysis from the perspective of statutory health insurance, expenditures will be determined on the basis of billing data.

The German version of the Involvement Evaluation Questionnaire (IEQ-EU) [11] will be used to assess the burden on informal caregivers.

To assess treatment satisfaction and subjective quality of life, the same instruments will be used for informal caregivers as for patients, specifically the ZUF8 [15, 16] and the WHO-QoL-BREF [17].

Dropouts and adverse events will be documented on standardised forms at the next follow-up assessment, at the latest.

Only trained GBV team members will administer the EPAS, CAN, and HoNOS and lead the enrolment procedure. A contact person at the coordinating centre will be available to GBV team members throughout the course of the study to answer questions on participant enrolment and screening. Refresher trainings will be offered as needed.

All local research workers have been trained and will follow uniform standard operating procedures (SOP) as to good clinical practice (GCP), recruitment, assessment, and questionnaires, for example. Advanced training sessions (e.g. training in HoNOS evaluation) of the research associates will be part of biannual project meetings. During the study, local research workers will be regularly tested for interrater reliability on the HoNOS and CAN using case vignettes.

Research workers at the coordinating centre will check the quality of data collection at each site during semiannual monitoring visits. The results will be documented in a monitoring report, which will be sent back to the local research workers and considered for the final analysis and interpretation of the data. The focus of the monitoring will primarily be on site monitoring, i.e. whether the local research workers are conducting the study in accordance with the given SOP. Furthermore, the monitoring will involve checking the completeness and plausibility of the available study documents. During the 1-year recruitment phase, the inclusion of study participants in accordance with the study protocol will be checked (e.g. by checking their eligibility and informed consent). Thereafter, the monitor will focus on the completeness and plausibility of the collected data and the documentation of serious adverse events. In addition, the monitor will offer support in case of questions or ambiguities in terms of participant recruitment, study conduct, and data collection and reporting, thus contributing to increased data quality.

### Plans to promote participant retention and complete follow-up {18b}

Participants will receive reimbursement for study participation (€25 per study visit, from *t*_0_ to *t*_4_) and, if applicable, for their expenses for travelling to the interview location.

### Data management {19}

Data will primarily be collected using the survey software SoSci Survey (SoSci Survey GmbH, Munich). The value ranges for the individual questions are defined in the electronic form of the questionnaire. A check for missing values is performed for each questionnaire in the electronic form. If not feasible, a paper version of the questionnaire will be used and transcribed into electronic format by the local research worker within 1 week. In order to check the data entry, staff at the coordinating centre in Ulm will re-enter a randomly chosen 5% sample of the paper-based data and subsequently compare the data sets.

All collected data will be regularly requested and stored on servers at the Department for Psychiatry and Psychotherapy II of Ulm University. Research workers at the coordinating centre at Ulm University will process and analyse the data. The data processing will include electronic data checks for plausibility and completeness as well as subsequent preparation for analysis. The data will be processed and evaluated with the software packages SPSS and SAS, among others.

The coordinating centre will oversee the whole study process by frequently communicating with local research workers regarding outstanding issues, checking the data collection procedure, and sending queries, if necessary.

### Confidentiality {27}

The collection, storage, and analysis of study data will be carried out in compliance with the relevant data-protection regulations. All personal data will be pseudonymised during the collection. The study participants’ personal identifying data will be replaced by identifiers. The index tables for the assignment of personal data and identifiers will be access-protected and stored separately from the data.

Communication and data transfer between the research team and both the local community mental health care providers and statutory health insurances will take place using screening identifiers. Local research workers will use study identifiers (different from screening identifiers) for data collection during the baseline and follow-up assessments (from *t*_0_ to *t*_4_). The local research workers will be the only persons with access to the major index table with personal data, screening identifiers, and study identifiers.

Prior to enrolment in the study, the potential study participants will be informed orally and in writing about the study’s aims, nature, scope, and implications by local GBV team members. In addition, the potential study participants will be informed about the data-protection regulations within the study. The potential study participants will be given sufficient time to think about their participation and to ask questions. By completing the informed consent form, the potential study participant will agree to participate in the study and to the data-protection regulations.

The study results will be published in anonymous form. This means that it will not be possible to allocate study data to individual study participants since no access to the index tables will be given.

The study data will only be transferred in anonymised form to third parties.

The study data will be archived on servers at the Department for Psychiatry and Psychotherapy II of Ulm University.

### Plans for collection, laboratory evaluation, and storage of biological specimens for genetic or molecular analysis in this trial/future use {33}

No biological specimens for genetic or molecular analysis will be collected in this trial.

## Statistical methods

### Statistical methods for primary and secondary outcomes {20a}

The data will be managed using IBM SPSS 25 and analysed using STATA 15 and SAS 9.4.

In order to evaluate the indication for GBV, the interrater reliability will be determined (using Spearman rank correlation) for the EPAS, HoNOS, and CAN ratings between screenings carried out by GBV team members and the baseline assessments carried out by local research workers. Furthermore, the criteria for serious mental illness will be assessed in the baseline data, and descriptive statistics will provide information on whether the target population has been recruited.

The analyses of primary and secondary outcomes will follow the intention-to-treat (ITT) principle using mixed-effects models with random time effects and time × treatment interaction effects. The covariance structure will take the study sites and individuals into account.

The cost-effectiveness ratios will be determined using the net benefit method, from the perspectives of both the statutory health insurance and national economy.

The implementation of GBV at the study sites will mainly be evaluated using descriptive methods. Mediator and moderator analysis will be conducted for the process variables, such as recovery orientation and multiprofessional collaboration.

### Interim analyses {21b}

No interim analyses are planned.

### Methods for additional analyses (e.g. subgroup analyses) {20b}

Diagnosis-specific effects will be examined using mixed-effect models extended by the interaction effect of time × treatment × diagnosis group. In addition, effects of GBV intensity on primary and secondary outcomes will be examined.. In-depth analysis should reveal effects of specific features of the GBV teams (e.g. peer-led teams, attitudes, recovery orientation, and interprofessional collaboration).

### Methods in analysis to handle protocol non-adherence and any statistical methods to handle missing data {20c}

In addition to the main analysis, a per-protocol (PP) approach will be followed to analyse the primary outcome in order to assess the maximal intervention efficacy in ideal conditions based on comparable outcome measurements. Criteria for the PP population will be defined in the statistical analysis plan prior to data analysis and may include

- More than 2 months between baseline assessment (*t*_0_) and initial meeting with the GBV team for participants in the intervention group
- Noncompliance with or infrequent use of GBV by participants in the intervention group
- Follow-up assessments (*t*_1_, *t*_2_, *t*_3_, and *t*_4_) being far outside the initially planned time periods (more than 2 months before or after the intended time points: 6 months, 12 months, 18 months, and 24 months after baseline assessment *t*_0_)

No imputation of missing data will be needed for the main analysis via mixed linear models. For the sensitivity analysis, missing values will be handled using the multiple imputations method [29]. Missing data will be imputed ten times. Then, each of the completed datasets will be analysed using the proposed statistical method. According to Rubin’s rule, the final result will be the average result of all completed datasets.

### Plans to give access to the full protocol, participant-level data, and statistical code {31c}

The full protocol (including the data-management and statistical-analysis plans) and the statistical code will be available on request from Prof Reinhold Kilian. Parts of the data sets will be published with open access according to data-protection regulations. More data will be accessible on request from Prof Reinhold Kilian.

## Oversight and monitoring

### Composition of the coordinating centre and trial steering committee {5d}

The team at the coordinating centre in Ulm comprises four persons:

RK, as the principal investigator, will oversee the entire study process. AMS will coordinate the study across sites and lead the data management and data analysis as a biostatistician. TB, as a psychiatrist, and FM, as a clinical psychologist, will address clinical issues in the study process, such as training the GBV team members and local research workers to use the assessment tools.

The trial-steering committee consists of RK, AMS, and TB from the coordinating centre and NG, EP, and JR from the Dachverband Gemeindepsychiatrie as the sponsoring organisation.

### Composition of the data monitoring committee, its role, and reporting structure {21a}

The team from the coordinating centre will monitor the data.

### Adverse event reporting and harms {22}

The study participants will be requested to immediately inform the local research worker (if possible) about serious adverse events. The occurrence of serious adverse events will be recorded, at the latest, at the next follow-up assessment. Subsequently, all serious adverse events will be reported to the coordinating centre in Ulm using standardised forms.

Community-based treatment, as intended in GBV, might lead to higher burden on relatives, and therefore, the burden on relatives was chosen as secondary outcome. No other adverse events are expected from trial participation.

### Frequency and plans for auditing trial conduct {23}

There are no plans for independent trial auditing.

### Plans for communicating important protocol amendments to relevant parties (e.g. trial participants, ethical committees) {25}

Any modifications to the protocol which may impact the study’s conduct or the potential benefit to the participants or which may affect participant safety will require a formal amendment to the protocol. Important protocol amendments must be submitted to local ethical committees and be communicated to all directly involved parties, specifically the umbrella association of community psychiatry (Dachverband Gemeindepsychiatrie), the local mental health care providers, the statutory health insurances, and the Institute for Epidemiology and Medical Biometry at Ulm University, which will randomise the participants. The entry at the German Clinical Trial Register DRKS00019086 is kept updated.

## Dissemination plans {31a}

The research results will be disseminated in peer-reviewed journals, together with anonymised data, where possible. Moreover, the results will be shared through oral and poster presentations at international conferences and at national stakeholder events. All project partners are encouraged to contribute to the dissemination of project outcomes. The researchers employed at the University of Ulm will lead the scientific dissemination of the study results. Authorship will provide credit for the researcher's contributions to the study and will carry accountability.

A project report in plain language will be sent to all participants and will be accessible on the project’s website. The only publication restrictions involve the national data-protection regulations.

## Discussion

This is the first RCT in Germany to examine the effectiveness and efficiency of integrated mental health care compared to CAU. This multicentre study will evaluate the impact of a community-based mental health care concept (GBV) on empowerment, quality of life, treatment satisfaction, and health economic measures among people suffering serious mental illnesses. The results of this study will help to decide whether GBV should be integrated into routine care for people with mental illness and financed by the statutory health insurance in the future.

A major strength of this study is the RCT design, which is recognised as the gold standard for examining the efficacy of interventions. High levels of academic rigour will be maintained throughout the data collection, data management, and data analysis by the involvement of independent, trained research associates and the use of standardised, validated scales. An additional strength is that the study will be conducted at different locations in Germany covering a broad range of catchment areas with different levels of urbanisation, different local mental health service systems, and different service providers. This will be advantageous when it comes to generalisation of the study results. In contrast, randomisation reduces the willingness to participate and therefore may lead to a selection effect. Another strong point of this study design is the economic evaluation from both national economic and statutory health insurance views. Patient reports on service use will provide the basis for estimating disease-related costs independently of funding agencies. In addition, health insurance claims data will be used to assess real costs for statutory health insurance.

The integrated care concept has been refined based on the results of the previous IVPOWER-study, which examined the efficacy of integrated care according to network mental health contracts. Fundamental changes were made with regard to participant enrolment and to the comparability of services across sites. An initial standardised assessment process regarding empowerment, unmet care needs, and psychosocial functioning was introduced to verify the need for such a comprehensive form of care by all community-based mental health service providers. Moreover, quality standards for GBV were set, and training activities across service providers were organised.

However, it is still to be assumed that service provision will vary across sites. As GBV is a complex intervention relying on good networking with local service providers, GBV depends on the available local service providers and their willingness to cooperate. Furthermore, it is not enough to define GBV’s individual components. Rather, the service providers’ processes and principles should be harmonised. Thus, differences in multiprofessional cooperation and the recovery orientation of GBV teams are assumed to impact GBV’s effectiveness. These aspects will be examined within the process evaluation, though the study is not designed to estimate site-specific effects. But when performing effectiveness analysis, the clusters of study sites will be taken into account in the covariance structure.

Another difficulty in this project is to avoid indirect changes in CAU by implementing GBV. GBV will be provided by teams working for community-based mental health service providers that offer a broad spectrum of services. Staff training and changes in infrastructure, especially communication channels, may also impact standard care. This applies especially to cases in which staff members are active in different services (e.g. GBV and outpatient sheltered living) at the same time. Nevertheless, this RCT should make it possible to demonstrate the added value of GBV, i.e. the additional care services such as case management, network discussions, and crisis services, even if the underlying standard care should improve in both groups.

## Trial status

Protocol version 1.0

Recruitment is planned to start on June 1, 2020, and is expected to last for 12 months until June 1, 2021.

## Data Availability

Parts of the datasets in anonymous form will be available from the corresponding author upon reasonable request. All data supporting the results reported in future articles will be included in anonymous form in these published articles and their supplementary information files.

## Abbreviations

CAN: Camberwell Assessment of Need—European version
CAU: Care as usual
CSSRI: Client Sociodemographic and Service Receipt Inventory
DGPPN: Deutsche Gesellschaft für Psychiatrie, Psychotherapie, Psychosomatische Medizin und Neurolgie (engl. German Society of Psychiatry, Psychotherapy, Psychosomatic Medicine and Neurology)
EPAS: Assessment of Empowerment in Patients with Affective and Schizophrenic Disorders
EQ-5D: Euro Quality of Life—5 dimensions
GBV: Gemeindepsychiatrische Basisversorgung (engl. community-based mental health care)
GCP: Good clinical practice
HoNOS: Health of the Nation Outcome Scale
IEQ-EU: Involvement Evaluation Questionnaire
ITT: Intention-to-treat
LPO: Last patient out
PP: Per-protocol
QALY: Quality-adjusted life years
RCT: Randomised controlled trial
SD: Standard deviation
SOP: Standard operating procedures
ZUF8: Questionnaire of Treatment Satisfaction; German version of the Client Service Questionnaire CSQ8
WHO-QoL-BREF: World Health Organisation Quality of Life—short version
